# New Insights on the Pathogenesis of Takayasu Arteritis: Revisiting the Microbial Theory

**DOI:** 10.3390/pathogens7030073

**Published:** 2018-09-06

**Authors:** J. Luis Espinoza, Suzue Ai, Itaru Matsumura

**Affiliations:** 1 Department of Hematology and Rheumatology, Kindai University Faculty of Medicine, 377-2, Ohno-Higashi, Osaka-Sayama, Osaka 577-8502, Japan; i.matsu@med.kindai.ac.jp; 2 Faculty of Medicine, Kindai University, 377-2, Ohno-Higashi, Osaka-Sayama, Osaka 577-8502, Japan; jlec19749@hotmail.com

**Keywords:** pulseless diseases, vasculitis, microbiome, molecular mimicry, autoimmune diseases, Tertiary lymphoid organ

## Abstract

Takayasu arteritis (TAK) is a chronic vasculitis that mainly affects the aorta, its major branches, and the pulmonary arteries. Since the description of the first case by Mikito Takayasu in 1908, several aspects of this rare disease, including the epidemiology, diagnosis, and the appropriate clinical assessment, have been substantially defined. Nevertheless, while it is well-known that TAK is associated with a profound inflammatory process, possibly rooted to an autoimmune disorder, its precise etiology has remained largely unknown. Efforts to identify the antigen(s) that trigger autoimmunity in this disease have been unsuccessful, however, it is likely that viruses or bacteria, by a molecular mimicry mechanism, initiate or propagate the auto-immune process in this disease. In this article, we summarize recent advances in the understanding of TAK, with emphasis on new insights related to the pathogenesis of this entity that may contribute to the design of novel therapeutic approaches.

## 1. Introduction

During the 12th annual meeting of the Japanese society of Ophthalmology held in Fukuoka, Japan, Mikito Takayasu, by then a professor of ophthalmology at Kanazawa University, presented what he described as “a case of peculiar changes in the central retinal vessels” in a young female patient with impaired vision [1,2].

Minoru Nakajima was the first to propose to coined the name “Takayasu disease” in 1921 when he reported several patients with symptoms resembling those described in Takayasu’s report [1,3]. Since then, several names, such as pulseless disease, aortic arch syndrome, obstructive productive arteritis, were proposed [2]. Following the publication in 1951 of an article, in which Japanese clinicians described various cases in an international journal, similar cases of this entity in other countries started to be reported [1,2,3]. In 1990 the American College of Rheumatology published the classification criteria (ARA criteria) for this disease and since then, the name “Takayasu arteritis” (TAK) was accepted worldwide [4].

Although it is well-known that TAK is associated with a profound inflammatory process, very likely caused by an autoimmune disorder, its precise etiology has remained largely unknown [5]. Nevertheless, in the last decade, substantial progress has been made in unraveling key pathogenic features of this disease [6]. In addition, novel biological and immunomodulatory agents, such as anti-TNF agents and tocilizumab, a monoclonal antibody against interleukin 6 receptor, have shown promising therapeutic potential in patients with TAK [7,8].

Remarkably, since vascular tissues are not exposed to the external environment, it has been historically believed that normal blood vessels are sterile, however accumulating evidence appear to indicate that indeed, there is a community of commensal microorganism in inner regions of human vasculature [9] and the composition of such “vascular microbiome” differs between healthy and pathologic vascular tissues and consequently, an altered microbiome, also known as dysbiosis, could be associated with the pathogenesis of inflammatory vascular diseases [10]. It is currently unknown if the presence of pathogenic microbes in the vascular tissues has a role in the development of TAK or if dysbiosis of the gut is implicated in the pathogenesis of this disease. In this article, we discuss current insights into the pathophysiology of TAK.

## 2. Takayasu Arteritis: An Overview

TAK is chronic inflammatory disease characterized by massive intima fibrosis and vascular narrowing which predominantly involves the aorta, its main branches, and pulmonary arteries [3]. Despite TAK primarily affecting young or middle-aged women, particularly from Asian countries, the disease has a worldwide distribution and has been reported in people of virtually all ages [11]. So far, the only genetic factor that shows a consistent association with TAK is the HLA allele, HLA-B*52, which has been confirmed in various cohorts and in several ethnicities [12,13,14]. Thus, it has been proposed that the increased prevalence of TAK in Asians may reflect the higher frequency of the HLA-B*52 in this population. An example of this is in the Japanese population, where the prevalence of TAK is 40 per million people, the frequency of HLA-B*52 is 10% [15]. Conversely, in most of the European population, where TAK is less prevalent, the frequency of HLA-B*52 is less than 2% [16].

In addition, genetic variants in non-HLA genes, especially in genes encoding immune response regulators and pro-inflammatory cytokines have been proposed as risk factors [5,17,18].

TAK can be divided into two phases: An initial acute phase (which is not evident in the majority of patients) of non-specific constitutional symptoms, including fever, malaise and weight loss [19]; and a second phase with symptoms suggestive of arterial occlusion and ischemia (pulselessness and bruits), claudication and hypertension [20]. If the diagnosis of TA is delayed, as frequently occurs, vascular inflammation can progress leading to stenosis, aneurysms and end-organ ischemia. In a recent report, the median age at death of patients with TAK was 38 (25–47) years, with vascular mesenteric ischemia and aortic aneurysm rupture the main causes of death [21].

Table 1 summarizes the most relevant demographic and clinical features of patients with TA.

Due to the unavailability of placebo-controlled randomized clinical trials, the management of TAK has relayed on results obtained from open studies, case series and expert opinion [30]. The most commonly used therapeutic agents include corticosteroids and conventional immunosuppressive agents such as methotrexate [30]. Novel therapeutic options, especially the use of biological agents, including agents against tumor necrosis factor α (TNF-α), rituximab (RTX) and tocilizumab (an inhibitor of interleukin 6 receptor IL-6R) in patients who do not respond to conventional immunosuppressive regimens have shown promising benefits [7,8], and the efficacy of this approach has been tested in randomized controlled trials recently [31,32]. Interestingly, the naturally derived stilbenoids curcumin and resveratrol, whose health-promoting properties have been extensively studied in preclinical models [33], were recently tested in two randomized trials in Chinese patients with TAK and the authors observed modest clinical responses in the interventional arms, compared with placebo groups that were attributed to the anti-TNF-α effects of these compounds [34,35] and likely to the properties of these agents to increase the proportion of circulating regulatory T cells (Treg) [36,37] since TAK patients have a reduced number of Treg cells [38,39].

## 3. Immunopathogenesis of TAK

**Figure 1:**
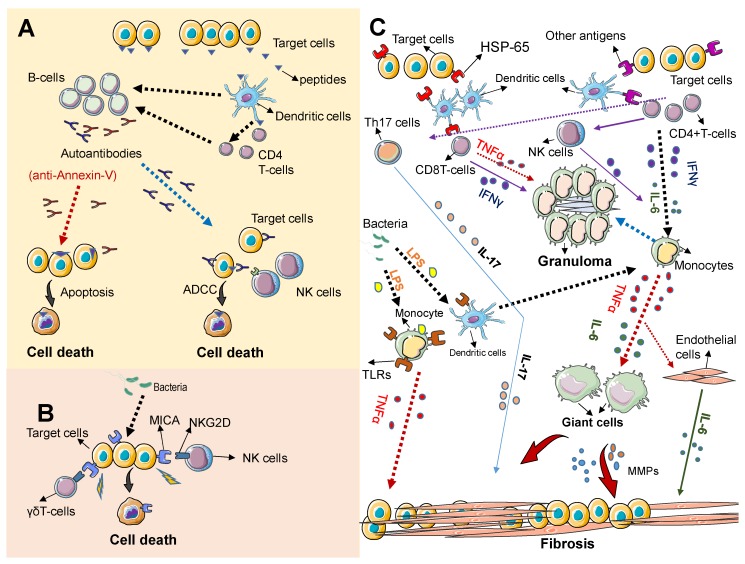
Current understanding of the pathogenesis of Takayasu arteritis.

As described above, TAK is characterized by the inflammatory damage of large blood vessels. The inflammatory process typically involves the inner wall and spares the outside of the blood vessels, progressing from a granulomatous inflammation (with infiltrating monocytes and lymphocytes) in early stages of the disease, to a less obvious inflammatory reaction in advances stages of the disease characterized by adventitial fibrosis, smooth muscle proliferation in the arterial intima and ultimately arterial stenosis [6,22]. Although the pathogenesis of TAK is not entirely known, the cause of this disease is likely rooted in a persistent inflammatory response from a genetically prone individual. Figure 1 summarizes the most relevant players in the pathogenesis of this disease.

An immune reaction against antigens, likely derived from microorganisms, either commensals or pathogens, is believed to be the initial event. (Figure 1A). Peptides derived from endothelial cells (target cells), likely resembling peptides derived from microorganisms and via antigen presenter cells (dendritic cells) are recognized as pathogenic by T-cells, stimulating B cells to produce specific antibodies that react against the patient’s own cells (autoantibodies). Some autoantibodies, such as anti-annexin-V directly induce apoptosis in endothelial cells and some may trigger apoptosis of target cells via antibody dependent cytotoxicity (ADCC) mediated by Natural killer cells (NK cells). (Figure 1B) Direct target cell death can be also mediated via NKGD/MICA interaction. Immune cells expressing the immune receptor NKG2D such as NK cells and γδT cells recognize the stress ligand MICA expressed on target cells leading to apoptosis of target cells. MICA can be induced by microbes or their products. (Figure 1C) Granuloma formation and tissue fibrosis result from the persistent activation of immune cells and the prolonged release of proinflammatory cytokines. A triggering antigen that mimics antigens expressed on normal cells (HSP-65) initiates an inflammatory cascade that involves the release of inflammatory cytokines and the activation of monocytes and other immune cells. Activated T cells release tumor necrosis factor alpha release (TNF-α) and IFNγ that promote prolonged monocyte activation, leading to granuloma formation. Persistently activated monocytes can also differentiate into giant cells, which promote vascular inflammation; and ultimately tissue fibrosis via matrix metalloproteases (MMPs) and TNFα secretion. The persistent activation of other immune cells, including Th17 and NK cells can also promote granuloma formation and fibrosis via the release of cytokines. This persistent inflammatory response can be initiated or propagated by bacteria derived products such as LPS that induce toll like receptor (TLRs) expression/activation on dendritic cells and monocytes. IL-6 secreted by various cellular sources, including immune cells and activated endothelia cells plays a crucial role in maintaining the inflammatory process that results in tissue fibrosis.

Compelling evidence strongly suggests that cell-mediated autoimmune responses play crucial roles in the pathogenesis of this disease, as indicated by the increased numbers of circulating T-cells observed in TAK patients, with most of the cells showing an activated state [6,38]. The underlying T-cell mediated autoimmunity is further supported by the reduced number of circulating Treg cells, a subset of T-cells required for maintaining immune homeostasis and prevention of autoimmunity, that have been documented in TAK patients [38]. In addition, T-cell receptor analysis of T-cells from TAK featured an oligoclonal profile [40] which is consistent with the notion that they proliferate in vivo in response to a specific antigen. The autoantigen eliciting such a T-cell activated state in TAK has remained elusive, however it is very likely that T-cells recognize antigens expressed on arterial tissues, as T-cells from TAK patients showed increased cytotoxicity against cultured human umbilical cord endothelial cells [41] and proliferated more vigorously in vitro in response to purified human aortal antigen extracts as compared to controls [42]. In addition, other immune cells infiltrating the inflamed vascular tissues of TAK include macrophages, γδ+ T cells and Natural Killer (NK) cells and B-cells [38,43].

The NK Group 2D (NKG2D) is an activating receptor expressed on NK cells and various subsets of T-cells [44] that recognizes specific ligands expressed on the surface of cells exposed to stress (infection or transformation) and therefore this receptor plays a crucial role in cancer immunosurveillance and immune response to various pathogens [45]. Intriguingly, an increase in Major Histocompatibility Class I Chain-Related A (MICA), which is a ligand for the NKG2D receptor, has been documented in aortic samples from TAK patients [46], suggesting that MICA engagement by NKG2D-expressing immune cells (NK or γδ-T cells) may contribute to the autoimmune response in this disease [45,47]. Interestingly, bacterial-derived stress signals capable of inducing MICA expression on normal intestinal tissues have been documented in the context of dysbiosis of gut microbiota in patients with inflammatory bowel disease, which contribute to the persistent inflammatory signals and autoimmunity observed in these conditions [45]. Further studies are needed to elucidate a potential involvement of dysbiosis in the induction of MICA in inflamed tissues of patients with TAK.

Furthermore, the possibility that specific immune response against microbe proteins cross-react with structurally related proteins of the host (molecular mimicry) has been proposed as the most plausible triggering mechanism [6]. Heat shock proteins (HSP), particularly 60 kDa HSP, constitute a likely target that may trigger an autoimmune response in TAK by this mechanism, due to its molecular analogy with the Mycobacterium tuberculosis (mTB) protein 65 kDa HSP [48].

In support of this assumption is the fact that an increased expression of 65 kDa HSP has been consistently found in aortic tissues of TAK patients [49], and specific T-cells and autoantibodies recognizing both human 60 kDa HSP and 65 kDa HSP have been observed in TAK patients [48].

Moreover, although B cells are not abundant in TAK lesions [43], an increased number of antibody secreting B cell subsets (CD19+/CD20−/CD27^high^) were found in the peripheral blood of patients with active TAK [50] and CD20+ B-cells surrounding the granulomatous lesions of TAK have been consistently documented [43], which together with the observations that some patients with active refractory TAK have shown excellent clinical response to the treatment with the anti-CD20 monoclonal antibody rituximab [50], indicating that B-cells may be involved in the pathogenesis of TAK. Recently, elevated levels of autoantibodies that react with endothelial cells were documented in the serum of patients with TAK and these pathogenic antibodies promoted the proliferation of endothelial cells in vitro by directly activating the mammalian target of rapamycin (mTOR) pathway. Notably, these effects were reversed when the cells were exposed to sirolimus, a specific inhibitor of mTOR [51], thus highlighting the therapeutic potential of blocking mTOR pathway in TAK.

In addition to the above described altered immune cell responses, elevated serum levels of various inflammatory cytokines such as interleukin-6 (IL-6), IL-8, IL-9, IL-17, IL-18 are frequently observed in TAK patients [52,53] and the serum levels of these factors, especially that of TNF-α and IL-6, often correlate with disease activity and decrease significantly in response to biological therapies [54] and novel inhibitors of TNF-α [55], as well as inhibitors of IL-6 receptor have shown promising clinical response in patients with TAK [56].

The elevated levels of cytokines detected in patients with TAK, may reflect the presence of an inflammatory process, regardless the triggering mechanism, however the release of certain cytokines may reflect the immune response against latent or active microbe infection. For example, mTB has been shown to induce IL-6 in vitro and in vivo [57,58] and TNF-α plays a critical role in the protective immune response against tuberculosis (TB) [59].

Not surprisingly, patients with latent TB infection that are treated with anti-TNF-α have increased risk to develop active TB infection [60]. Interestingly, a recent meta-analysis reported that a polymorphism in the TNFα gene (TNFα308A/G) is associated with susceptibility to TAK [13]. Individuals with the GG genotype secrete less TNFα and have higher risk to develop TAK compared with individuals with the AA genotype [13]. To explain those paradoxical findings, it has been proposed that individuals carrying the GG genotype are less competent (secreting less TNFα) than those with the GA or AA genotype in mounting an immune response against mTB and eliminating it from the body, which in turn could result in the persistence of the bacteria antigen predisposing individuals with GG allele to autoimmunity [61].

In a recent study, elevated plasma levels of pentraxin-related protein (PTX-3) were reported in TAK patients. Although PTX-3 levels did not associate with disease activity, PTX-3 levels significantly correlated with serum CRP levels [62]. PTX-3 is an inflammatory protein rapidly released by several cell types including monocytes, dendritic cells and endothelial cells in response to primary inflammatory signals such as toll-like receptor (TLR) engagement and TNF-α activation. PTX-3 acts as a secreted pattern-recognition receptor that directly interacts with pathogenic microorganisms such as *Aspergillus fumigatus* [63], *Neisseria meningitides* [64] and *Pseudomona aeruginosa* [65] and thus inflammatory signals triggered by PTX-3 may support the involvement of either pathogenic microbes or commensal microorganisms in the pathogenesis or progression of TAK. Further well-designed studies are needed to clarify a potential link of gut microbiota composition with PTX-3 levels in healthy individuals and in patients with TAK or with other vasculitides.

## 4. Infections and TAK

A link between TAK and infections has long been considered. In fact, in his case report, Takayasu emphasized that he and his colleagues did not find evidence of infections, such as tuberculosis (TB) or syphilis [1]. A putative association between TAK and latent and active TB was first proposed based on the findings of granulomas-like lesions in affected arteries of patients with TAK, which histologically resembled granulomas found in tuberculosis [1]. Further epidemiological and clinical studies reporting higher frequency of TB in patients with TAK, compared with the general population, also suggest a possible causal association between these two diseases [66]. Moreover, the finding of Rasmussen’s aneurysms in inflamed arteries near tuberculosis cavities in the lung parenchyma of patients with pulmonary TB [67], together with the high frequency of tuberculin skin test positivity in TAK patients [6] further support this hypothesis. A more suggestive link between TB and TAK was the discovery that IS6110 and HupB gene sequences of *mTB* are frequently detectable in formalin fixed aortic specimens of patients with TAK, indicating the possibility that the vascular lesions of TAK could be a clinical manifestation of extra-pulmonary tuberculosis [68].

Nevertheless, other studies failed to demonstrate a link between TB and TAK. For example, in a study using using the Quantiferon-TB Gold test (QFT), an in vitro assay measuring interferon-gamma (IFN-γ) response to mTB antigens and helpful in diagnosing latent TB infection, QFT positivity was similar between TAK patients and controls [69]. It must be noted, however, that in the same study, tuberculin positivity was higher in the TAK group than in controls and whereas only one control had a history of previous TB infection, six TAK patients had previous TB history. In addition, it is unknown if the fact that TAK patients were older that control may have affected the IFN-γ response in this study [69]. Moreover, Arnaud et al. did not detect the presence of *mTB* complex RNA in arterial lesions of fresh arterial samples obtained from TAK patients, after being assessed by three different methods: Acid fast and auramine-fluorochrome staining, mycobacterial cultures, and nucleic acid amplification. Although the authors did not rule out the likelihood of a cross-reaction between mycobacterial and arterial antigens [70]. Similarly, whereas IS6110 sequence was found in peripheral blood from 78% patients with pulmonary tuberculosis, all samples from patients with TAK were negative for the insertion sequence IS6110 and for HSP65 and 16S rRNA genes [71]. The above data indicate that although there are various pieces of evidence that implicate mTB with TAK, confirmatory studies are needed to establish a direct causal association between this pathogen and TAK. Future studies, using large number of patients are needed to establish the existence of causal relationship between TAK and mTB.

In addition to the proposed causal role of mTB, other microorganisms including *streptococcus* and *spirochetes* have been investigated in association with TAK, although there is no convincing evidence to support a potential association of any of these pathogens with TAK [72].

Intriguingly, in some patients, TAK is associated with sarcoidosis [28,73], a multisystem disorder of unknown etiology characterized by non-necrotizing granulomas composed of infiltrating T-lymphocytes, mononuclear phagocytes in affected tissues. Although the etiology of sarcoidosis is largely unknown, the involvement of a pathogen-triggered autoimmune reaction has been proposed [74,75] and thus the concurrence of both diseases in some patients suggest the potential existence of overlapping mechanisms in the pathogenesis of these diseases [65]. On the other hand, the coexistence of TAK with ulcerative colitis, an inflammatory bowel disease associated with particular genetic background and alterations in the gut microbiota composition, has been frequently reported in the literature [76,77].

*Chlamydia Trachomatis* was recently documented in patients with cutaneous vasculitis and TAK [78], however, it is unclear if those findings are representative of TAK or if they are an atypical finding since patients reported in that study also presented with Cogan’s syndrome [78].

In preclinical models of vasculitis, dendritic cells at the media-adventitia junction of medium and large arteries were identified as dominant pathogen sensors that recognize stimuli via TLR2 and TLR4 pathways that ultimately render the vessel wall susceptible to immunological attack mediated by T-cells leading to an inflammatory process that resembles giant arteritis or TAK [79,80] suggesting that microbe-induced inflammatory stimuli may trigger the autoimmune reaction in these disorders.

Early studies reported the induction of vasculitis with chronic elastic arteritis resembling TAK lesions in gammaherpesvirus 68 (MHV-68)-infected mice [81]. MHV-68 is homologous to human pathogenic herpesvirus, including Epstein Barr virus (EBV) and KSHV. Interestingly, chronic or active EBV infections have been reported in patients with TAK, especially in pediatric patients [82]. Given the ubiquitous distribution of EBV, infecting more than 90% of adults worldwide [83], if the virus is implicated in the pathogenesis of TAK, it is likely that certain factors inherent to the host, such as genetic predisposition, the exposure to certain environmental factors or the coexistence of other pathogens, are required for disease induction, which would explain the higher incidence of TAK in the Asian population. To support this notion, a link between EBV and other diseases in certain populations has been consistently demonstrated, including the high prevalence of nasal NK/T lymphoma in Asia and Latin America and Burkitt’s lymphoma in south Saharan African children [83].

## 5. Is Microbiota Implicated in the Pathogenesis of TAK?

The commensal organisms (bacteria, viruses, and fungus) that colonize human body niches, including mouth, nose, skin, and gut are collectively known as microbiota [84]. Gut microbiota plays crucial roles in the development and education of immune system [84,85] and can regulate hematopoiesis [86]. Not surprisingly, and altered microbiota composition has been linked to various human disorders ranging from autoimmune diseases and cancer [87,88].

In addition, the presence of microbes within normal or diseased blood vessels has been also reported. For example, herpes simplex virus (HSV), EBV and *cytomegalovirus* (*CMV*) were detected in atherosclerotic plaques and in unaffected bypass grafts [89] and *Mycoplasma pneumoniae* (*M-pneumoniae*)-specific DNA was detectable with similar frequency in atherosclerotic plaques and normal saphenous veins tissues [90]. Similarly, various microorganisms including *helicobacter pylori*, HSV, *C-pneumoniae*, *CMV*, and *M-pneumoniae* were detectable in normal aortic wall tissues during coronary artery bypass or aortic valve replacement procedures [91].

A recent study revealed that the gut microbiota composition of patients with Kawasaki disease, a febrile acute vasculitis that predominantly affects infants and presents with mucosal and skin manifestations [9], strongly correlates with disease status [92].

Though various studies have explored potential associations between the microbiome and various vasculitides [10], so far there are no reports on the gut microbiome composition in patients with TAK. Ethical and technical issues for obtaining optimal tissue samples from larger vessels and the fact that TAK is a rare disease, may account for the unavailability of microbe analysis data in this disease.

Recent data supporting the existence of specific oral and gut microbiota signatures in patients with Behcet disease, a systemic inflammatory condition with ocular manifestations and mucocutaneous and vascular involvement [93,94], are encouraging since interventional approaches aimed to modify the gut microbiota composition in selected patients, for example by using probiotics or fecal microbiota transplant, may have promising therapeutic potential in these disorders.

As mentioned above, it is likely that microbe-derived products, either from commensals or pathogenic microorganism, may trigger autoimmunity in TAK via molecular mimicry, in which the sequence similarities between foreign and self-peptide results in cross-activation of pathogen-derived autoreactive T or B cells leading to autoimmunity [95]. In this regard, antigens derived from microbes, for instance mTB would trigger autoimmunity in a genetically predisposed host (individuals harboring HLA-B*52) ultimately leading to TAK. This mechanism is clearly exemplified by commensal-induced autoimmunity observed in patients with rheumatoid arthritis in which *N*-Acetyl-glucosamine-6-sulfatase and filamin A proteins derived from gut bacteria (*Prevotella* sp. *Butyricimonas* sp and *Parabacteroides* sp.) were identified as autoantigens that produce responses from both T and B cells, in more than 50% of patients, but not in healthy controls or patients with other rheumatic diseases [96].

**Figure 2:**
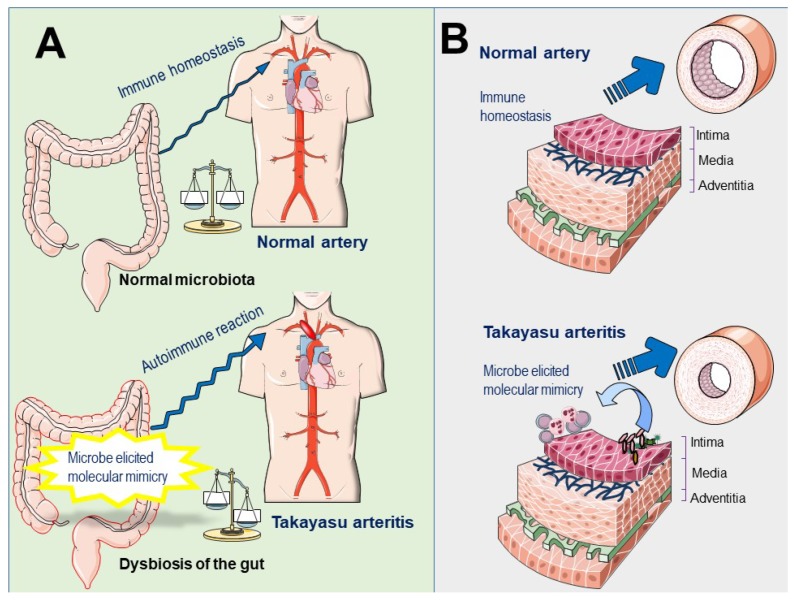
Potential role of pathogenic or commensal microbes in the pathogenesis of Takayasu arteritis. (**A**) Gut microbiota. A normal gut microbiota contributes to the development and education of the immune system, which is essential for the maintenance of physiologic homeostasis. Conversely, in the presence of dysbiosis, the number and function of regulatory T-cells are impaired and hyperactive cytotoxic T cells may grow without control, consequently the immune equilibrium is lost. The growing of pathogenic bacteria in the gut, likely harboring antigens that resemble antigens expressed by aortic or vascular tissues of the host, may promote the sensitization of the immune cells to the host own antigens, via molecular mimicry leading to autoimmunity. (**B**) Vascular microbiota. The colonization of pathogenic bacteria in aortic tissues may promote a local immune reaction against pathogen proteins and recruited immune cells may cross-react with antigens expressed in normal tissues leading to autoimmunity.

Tertiary lymphoid organs (TLOs) are ectopic lymphoid structures developed at sites of chronic inflammation associated with infection or autoimmune disorders [97]. TLOs have been recently documented in the aortic walls of patients with active TAK. This ectopic neogenesis is composed of a high percentage of memory and antigen-experienced CD4+ T cells that orchestrate B-cell activation, and antigen-driven clonal expansion [98]. Importantly, dysbiosis-induced TLO was recently reported to aggravate the severity of inflammatory bowel disease [99], supporting the role of microbiota dysregulation in the development of autoimmunity and suggest the potential involvement of gut dysbiosis or local dysbiosis of vascular microbiota in the development of TLO in patients with TAK (Figure 2).

## 6. Concluding Remarks and Future Directions

TAK is a rare vasculitis affecting the aorta or its principal branches. Technical and ethical issues are important limitations that impede obtaining suitable arterial samples from living patients with TAK for the study of this disease. Although the pathogenesis of this disease has not been clarified, it is well-known that an immune reaction targets the arterial wall, leading to progressive wall fibrosis, which ultimately results in luminal stenosis or aneurysmal formation. A crucial role of autoimmunity in the etiology of this entity is demonstrated by a good clinical response observed in most patients treated with corticosteroids or immunosuppressive drugs. In addition, several effectors that play important roles in the inflammatory process of this disease have been identified, including lymphocyte subsets, macrophages and proinflammatory cytokines (IL-6, IL-17, IFNγ and TNFα). However, the antigen(s) that trigger such autoimmune reaction remain elusive. Identifying the triggering mechanism is essential not only to improve the immunomodulatory therapies for this disease but also for the possibility that interrupting the eliciting agent can revert or attenuate the inflammatory process that leads to vascular stenosis.

An association of microorganisms with TAK has been extensively investigated and, so far, based on epidemiological and laboratory data, the most likely pathogen that appears to be implicated in the pathogenesis of TAK is mTB. Surprisingly, while mTB can infect mice, and various mouse models of tuberculosis have been reported [100,101], to the best of our knowledge, there are no studies on the effects of this pathogen on the mouse vasculature and therefore, it is currently unknown if mTB can cause TAK-like changes in the infected mice. Colonization of vascular tissues by pathogenic or commensal microbial agents, has been documented in vascular diseases, especially in atherosclerosis, aneurysms, and more recently in Behcet disease. In addition, recent evidence indicates that healthy blood vessels may harbor their own commensal microbiome, and it is likely that vascular dysbiosis may account for the chronic inflammation occurring in systemic vasculitides, however, as mentioned above, the study of such a “vascular microbiome” in patients with TAK is challenging due to the challenges of obtaining suitable specimens. Nevertheless, gut microbiota is more accessible due to the relative simplicity of acquiring samples and the extensive experience in the analysis of its composition. It is expected that ongoing studies investigating the gut microbiome composition in patients with TAK will likely shed some light on a putative implication of microbes with the pathogenesis of this disease.

## Figures and Tables

**Table 1:** Demographic characteristics and main clinical features of Takayasu arteritis.

Demographic Data		Ref
Predominant gender:	Female (80%)	[11,22,23]
Age at onset (years)	23 (0–65)	[11,22,23]
Ethnicity	Rare in western countries (0.4–3 per million people). More common in southeast Asia, Japan, China, India. (40 per million in Japan)	[11,22,23,24]
**Clinical Findings**	* **Frequency (%)**	
**Constitutional Manifestations**		[19,25,26,27]
Malaise	29–56	
Fever	17–32	
Anorexia	15–34	
Weight loss	20–25	
**Cardiovascular Findings**		[19,22,25,26,27,28]
Hypertension	33–56	
Bruit of carotid arteries	32–62	
Claudication	30–80	
Dyspnea	10–50	
Carotidynia	10–36	
**Central Nervous System**		[19,25,26,27,29]
Headache	50–70	
Dizziness/vertigo	24–55	
Syncope	4–19	
Visual disturbances	15–35	
Stroke	3–22	
**Skin and Musculoskeletal**		
Myalgia	30	[25]
Arthralgia	28–39	[19,22,25]
Synovitis	7.7	[25]
Skin rash	7.8–20	[19,26]

* The frequency of the clinical findings varies depending on the reference consulted and may be determined by differences in data collection, patient assessment, the geographic precedence of the study and whether the reported cases were hospitalized patients or outpatients.
